# Management of Subutex Addiction: A Case Report in a tunisian psychiatry unit

**DOI:** 10.1192/j.eurpsy.2025.984

**Published:** 2025-08-26

**Authors:** A. Rami

**Affiliations:** 1 Razi Hospital, Manouba, Tunisia

## Abstract

**Introduction:**

Subutex abuse in Tunisia is a phenomenon that is becoming more and more frequent and the management of which is not obvious due to the development of this substance in parallel markets.

**Objectives:**

Our objective was to assess the patient’s Subutex use and initially assist with withdrawal, followed by achieving abstinence.

**Methods:**

We followed the case of a patient in the psychiatric unit B at the Razi hospital, for depressive symptoms and having a problem of misuse of Subutex for 4 years, in a difficult family and social context.

**Results:**

Patient follow-up was done over a one-year period with weekly consultation sessions at the beginning to assess the situation. Although withdrawal was done after a few weeks, it was essential to maintain this abstinence, using psychotherapy based on CBT done over 7 sessions.

During the study period, the patient violated abstinence only once in a family conflict.

**Conclusions:**

The patient’s response to our management has reinforced the hope of being able to treat addiction to Subutex despite the severity of his condition and family and social instability.

**Disclosure of Interest:**

None Declared

